# Chronic Timed Sleep Restriction Attenuates LepRb-Mediated Signaling Pathways and Circadian Clock Gene Expression in the Rat Hypothalamus

**DOI:** 10.3389/fnins.2020.00909

**Published:** 2020-09-08

**Authors:** Qi Sun, Yang Liu, Wei Wei, Dan Wu, Ren Lin, Deliang Wen, Lihong Jia

**Affiliations:** ^1^Department of Child and Adolescent Health, School of Public Health, China Medical University, Shenyang, China; ^2^Institute of Health Science, China Medical University, Shenyang, China

**Keywords:** sleep restriction, appetite, hypothalamus, leprb signal pathway, circadian clock

## Abstract

The sleep duration of adolescents has continued to decline over the past 20 years. Sleep insufficiency is one of the most important risk factors for obesity, but the mechanisms underlying the association are unclear. Therefore, the hypothalamic-regulated mechanisms of appetite and the circadian clock gene expression were examined in sleep-restricted rats. Rats aged 7 weeks were randomly divided into two groups: the control group and sleep restriction group (7 rats/group) rats were sleep-restricted for 4 weeks. Body weight gain and amount of food/water consumption were quantified. The expression of genes or proteins which regulated appetite and energy metabolism via leptin receptor signaling and the circadian clock in the hypothalamus were assessed. Chronic sleep restriction induced increased food intake and weight gain in adolescent and young adult rats from the second week of initiation of sleep restriction. Phosphorylation of Janus kinase 2 (JAK2)/signal transducer and activator of transcription 3 (STAT3) was decreased, although levels of circulating leptin or leptin receptor expression were unaltered. Furthermore, insulin receptor substrate (IRS)/phosphoinositide 3-kinase (PI3K)/AKT/mTOR and forkhead box O1 (FoxO1) signaling pathways were also compromised. Moreover, core circadian clock genes were also decreased in the sleep restriction group compared with the control. Chronic timed sleep restriction induced hyperphagic behaviors, attenuated leptin receptor-mediated signaling pathways, and depleted the expression of circadian clock gene in the hypothalamus of adolescent and young adult rats.

## Introduction

Insufficient sleep in children and adolescents is highly prevalent worldwide. It is reported that 72.7% of high school students and 57.8% of middle school students in the United States do not get enough sleep (Wheaton et al., 2018). Longitudinal studies have provided evidence that children who sleep for a shorter duration are more likely to have a higher body mass index when they are adults (Landhuis et al., 2008). The risk of obesity in adolescent males who slept less was shown to be 1.8 times higher in a cross-sectional study (Suglia et al., 2014). Our previous study also reported that shorter sleep durations might increase the risk of being overweight or obese in different age groups of students (Sun et al., 2018). Several studies have reported that sleep curtailment might affect children’s self-control when making food choices, resulting in increased intake of unhealthy foods and increased risk of overeating (Hjorth et al., 2014; Hibi et al., 2017; Martinez et al., 2017). The hypothalamus in the central nervous system plays an essential role in the modulation of appetite and energy expenditure. Compromised hypothalamus regulation is involved in the development of obesity (Carmo-Silva and Cavadas, 2017; Marraudino et al., 2019; Seong et al., 2019). However, the effect of sleep curtailment on the regulation of appetite in the hypothalamus and the mechanisms underlying this are not fully understood and need further exploration.

Leptin, secreted by peripheral adipose tissue, connects the circuit of endocrine feedback between the hypothalamus and peripheral signals (Wasim et al., 2016). The long form of the leptin receptor (LepRb) expressed in hypothalamic neurons binds to leptin found in plasma under physiological conditions. LepRb then associates with the cytoplasmic tyrosine kinase Janus kinase 2 (JAK2), which induces downstream signals. Signal transducer and activator of transcript 3 (STAT3) is phosphorylated by LepRb-mediated JAK2, which then translocates into the nucleus and binds to the promoter of the anorexigenic proopiomelanocortin (POMC) gene. This reduces food intake and increases energy expenditure (Barrios-Correa et al., 2018). Suppressor of cytokine signaling-3 (SOCS3) is also a target gene of STAT3, which can dephosphorylate JAK2 to terminate leptin signal transduction (Engin, 2017). It has been demonstrated that chronic sleep deprivation can attenuate the phosphorylation of STAT3 in the hypothalamus of mice (Hakim et al., 2015), but the mechanisms require further exploration.

Epidemiological and experimental studies have shown that sleep deprivation, shift work, and jet lag may cause circadian clock disruption (Cedernaes et al., 2015; Parsons et al., 2015). It is widely acknowledged that the circadian rhythm can control energy metabolism (Stenvers et al., 2019). The circadian rhythm, or so-called circadian clock, establishes a daily 24 h sleep-wake alternating cycle. The master clock located in the suprachiasmatic nucleus (SCN) of the mammalian’ hypothalamus is dampened by transcriptional feedback circuits formed by core clock genes including *Clock*, *Bmal1*, *Per1*, *Per2*, *Cry1*, and *Cry2*. Previous studies have demonstrated that cortical expression of several clock genes is affected by sleep deprivation (Leng et al., 2019). However, the effects of sleep deprivation/restriction on clock genes in the hypothalamus are not entirely clear.

Moreover, SCN can make projections to different types of cell bodies in the hypothalamus, including the arcuate nucleus (ARC), where neurons express POMC and orexigenic neuropeptide Y from reciprocal connections within neural circuits located in the SCN (Huang et al., 2011). Therefore, we hypothesized that the changes of clock genes and LepRb-mediated signaling pathways could be intrinsically linked. Our study was designed to investigate the effects of chronic sleep restriction on leptin receptor signaling pathways and circadian clock expression in the hypothalamus of adolescent to young adult rats, illuminating the mechanisms associating sleep curtailment and obesity.

## Materials and Methods

### Animals

Sprague-Dawley rats weighing between 250 and 270 g purchased from the Experimental Animal Center of China Medical University were used in this study. All procedures were approved by the Animal Use and Care Committee of China Medical University, abiding by the National Institutes of Health Guide for Care and Use of Laboratory Animals. The animal room was maintained at 22–24°C, with a 12 h light/dark cycle (light on 8:00 a.m. to 8:00 p.m.) and a relative humidity of 55–65%. Male rats aged 6 weeks were housed (three or four per cage) in sterilized plastic cages with wood shaving bedding. The experiment began after 1 week of adaptive feeding.

### Reagents

TRIzol reagent was obtained from Takara (Japan). RIPA Lysis Buffer was purchased from Beyotime Biotechnology (China). The enhanced chemiluminescence (ECL) plus kit and bicinchoninic acid (BCA) protein assay kit were obtained from Pierce (Thermo Fisher Scientific, United States). The primary antibodies against LepRb (sc-842), phosphorylated-JAK2 (p-JAK2, Tyr1007/Tyr1008, sc-16566), JAK2 (sc-278), POMC (sc-20148), and SOCS3 (sc-73045) were purchased from Santa Cruz Biotechnology (United States). The primary antibodies against phosphorylated-STAT3 (p-STAT3, ^#^52075), STAT3 (^#^30835) and phosphorylated-insulin receptor substrate (p-IRS1, Ser636/639, ^#^2388), phosphoinositide 3-kinase (PI3K, ^#^4292), phosphorylated-BMAL1 (p-BMAL1, Ser42, ^#^13936S), BMAL1 (^#^14020S), phosphorylated-AKTT (p-AKT, Thr308, ^#^13038), phosphorylated-AKTS (p-AKTS, Ser473, ^#^4060), AKT (^#^4685), phosphorylated-mammalian target of rapamycin (p-mTOR, Ser2448, ^#^2971), mTOR (#2972), phosphorylated-forkhead box O1 (p-FoxO1, Thr24, ^#^9464), FoxO1 (^#^2880), IRS1 (^#^2382), and β-actin (^#^4970) were procured from Cell Signaling Technology (MA, United States). The primary antibodies against CLOCK (^#^ab3517) and phosphorylated-PI3K (p-PI3K, Y607, ^#^ab182651) were purchased from Abcam (Cambridge, MA, United States). The primary antibodies against phosphorylated-IRS2 (p-IRS2, Ser1100, ^#^AF8383), IRS2 (^#^DF7534), PER1 (^#^AF4602), and PER2 (^#^AF4601) were purchased from Affinity Biotechnology (Changzhou, China). The primary antibodies against CRY1 (^#^A13662) and CRY2 (^#^A6891) were purchased from ABclonal (Wuhan, China). All other chemicals were of analytical grade and obtained from the local chemical suppliers.

These chemical reagents were prepared as stock solutions with sterile water and then diluted to the final concentrations before application. Water used in this study was double distilled.

### Sleep Restriction

The rats were randomly divided into the control and sleep restriction groups after adaptive feeding and gentle handling for 1 h each day for 1 week. Sleep-restricted (SR) rats were handled each time they showed physical signs of sleepiness (no motor activity). The gentle handling procedures included touching their fur and whiskers with a cotton tip applicator or gently tapping on their cages. During the course of this study, to mimic the chronic sleep deficiencies observed in humans, the rats in the sleep restriction group were gently handled for 4 h each day (8:00 a.m.–12:00 p.m.) for 4 weeks, while the control groups was left to sleep without any disturbances.

### Body Weight

The body weight of the animals was measured twice a week for 4 weeks (7 rats/experimental group). Each measurement was taken at the same time during the middle of the light/dark cycle. Body weight gain was calculated by subtracting the body weight determined by the day before the first day of sleep restriction, from body weight on exposure days.

### Food Intake and Drinking Water

During the study, *ad libitum* access to standard chow and normal water was available to the animals. Food and water intake was measured daily by calculating the amounts of food and water consumed in each cage and dividing it by the number of animals in the cage. Food intake was presented as g/day/rat, and drinking water was presented as mL/day/rat.

### Western Blotting

All animals were sacrificed at the end of sleep restriction. Samples of the hypothalamus were taken immediately and stored at −80°C until analysis. Total protein samples were extracted from the hypothalamus using RIPA lysis buffer. Protein concentrations in the lysates were determined by a BCA protein assay kit. Aliquots of each sample (50 μg per lane) were loaded on to 8% SDS-polyacrylamide electrophoresis gels. Blots were transferred onto polyvinylidene difluoride (PVDF; Millipore, Bedford, MA, United States) membranes electronically. The membranes were blocked with 5% BSA for 2 h at room temperature followed by probing with primary antibodies against LepRb (1:500, rabbit anti-rat); p-JAK2 and JAK2 (1:500, rabbit anti-rat); p-STAT3 (1:500, rabbit anti-rat); STAT3 (1:1000, rabbit anti-rat); SOCS3 (1:500, rabbit anti-rat); POMC (1:500, rabbit anti-rat); p-IRS1, p-IRS2, p-PI3K, p-AKTS, p-AKTS, p-mTOR, and p-FoxO1 (1:500, rabbit anti-rat); IRS1, IRS2, PI3K, AKT, mTOR, and FoxO1 (1:1000, rabbit anti-rat); p-BMAL1 and CLOCK (1:500, rabbit anti-rat); BMAL1, CRY1, CRY2, PER1, and PER2 (1:1000, rabbit anti-rat); and β-actin (1:2000, rabbit anti-rat) as a housekeeper at 4°C overnight. The bands were detected via a secondary horseradish peroxidase (HRP)-conjugated anti-rabbit IgG antibody for 1 h at room temperature. The specific bands were visualized using an ECL Plus kit. Membranes were imaged by an Azure c300 Imaging System (Azure Biosystems, Dublin, CA, United States). Semi-quantification was performed using Image-Pro Plus 6.0 software (Media Cybernetics, ML, United States), normalized to β-actin levels from the same blot, and presented as a fold change vs. the control sample.

### Real-Time RT-qPCR

Quantitative real-time PCR (RT-qPCR) was conducted according to the Minimum Information for Publication of Quantitative Real-Time PCR Experiments (MIQE) guidelines. Total RNA was extracted from the hypothalamus with TRIzol reagent. The cDNA was synthesized from total RNA using a PrimeScript RT reagent kit (Takara, Japan) and served as a template for real-time PCR amplification. SYBR Green (Takara, Japan) and QuantStudio 6 Flex Real-Time PCR System were used for PCR (Life Technologies). To amplify the fragments of JAK2, STAT3, SOCS3, IRS1, IRS2, PI3K, AKT, mTOR, FoxO1, POMC, BMAL1, CLOCK, CRY1, CRY2, PER1, and PER2, the specific primer pairs detailed in Table 1 are used. Amplification was conducted for 40 cycles of 5 s at 95°C and 34 s at 60°C. Results were analyzed using the comparative Ct method as described by Livak and Schmittgen (2001). RNA abundance was expressed as 2^–ΔΔ*CT*^ for the target gene normalized against GAPDH (as the housekeeper gene) and presented as fold change vs. control sample.

**TABLE 1:** Oligonucleotide sequences used for real-time RT-PCR.

Gene	Primer sequences	Product (bp)
LepRb	5′-TCTTCTGGAGCCTGAACCCATTTC-3′	675
	5′-TTCTCACCAGAGGTCCCTAAACT-3′	
JAK2	5′-TTTGAAGACAGGGACCCTACACAG-3′	101
	5′- TCATAGCGGCACATCTCCACA-3′	
STAT3	5′-CACCCATAGTGAGCCCTTGGA-3′	137
	5′-TGAGTGCAGTGACCAGGACAGA-3′	
POMC	5′-CATAGACGTGTGGAGCTGGT-3′	149
	5′-TCAAGGGCTGTTCATCTCCG-3′	
SOCS3	5′-AGTCGGGGACCAAGAACCTA-3′	172
	5′-GAAGGTTCCGTCGGTGGTAA-3′	
IRS1	5′-AGAGTGGTGGAGTTGAGTTG-3′	277
	5′-GGTGTAACAGAAGCAGAAGC-3′	
IRS2	5′-GACTTCTTGTCCCATCACTTGAAA-3′	112
	5′-GCTAAGCATCTCCTCAGAATGGA-3′	
PI3K	5′-GCCCAGGCTTACTACAGAC-3′	248
	5′-AAGTAGGGAGGCATCTCG-3′	
AKT	5′-CAAGATGACAGCATGGAGTGTG-3′	189
	5′-CCAGCACATCCGAGAAACAAAA-3′	
mTOR	5′-GACAACAGCCAGGGCCGCAT-3′	154
	5′-ACGCTGCCTTTCTCGACGGC-3′	
FoxO1	5′-TCCTCGAACCAGCTCAAACG-3′	288
	5′-GGCGGTGCAAATGAATAGCAAG-3′	
CLOCK	5′-CTGTTACATCAGCACGCCTC-3′	145
	5′-TACTGCTTGGCTCTTCTGCT-3′	
BMAL1	5′-CCGATGACGAACTGAAACACC-3′	78
	5′-TCTTCCCTCGGTCACATCCT-3′	
CRY1	5′-ACGTGATAGGGAAGTGCACA-3′	135
	5′-GTGCCTCAGTTTCTCCTCCT-3′	
CRY2	5′-ACCGCCTGTGGGACTTGTA-3′	80
	5′-TCGCCATAGGAGTTGTCCAAATA-3′	
PER1	5′-GCTCCTGACCAAGCCTCATT-3′	165
	5′-GCAGGAAGAGGAGGCACATT-3′	
PER2	5′-CTGCGAAGCGCCTCATTCC-3′	295
	5′-TTATGCTCCGCCTCTGTCATC-3′	
GAPDH	5′-GCAAGAGAGAGGCCCTCAG-3′	74
	5′-TGTGAGGGAGATGCTCAGTG-3′	

### Serum Leptin Level

Leptin level was assessed in serum obtained directly from the abdominal aorta at the end of sleep restriction exposure (12:00 pm) via a fast-surgical procedure following diethyl ether anesthesia. Blood was centrifuged, and serum was frozen at −80°C. The leptin level in the serum was assessed using commercially available ELISA Kits (Quantikine ELISA Kit R&D Systems, Minneapolis, MN, United States) according to the manufacturer’s instructions. The leptin level was calculated using an appropriate standard curve generated by the ELX800 ELISA plate reader (BioTek Instruments, United States). The results were expressed as ng/mL.

### Statistical Analysis

All data were expressed as the mean ± standard deviation (*SD*). Significant differences among group means were calculated using SPSS v22.0 (SPSS Inc., United States). Repeated-measure analysis of variance (ANOVA) was used to compare food intake and weight gain, and comparison of all other quantitative data was performed using independent-sample *t*-test applied in this study. Statistical significance was considered as *p* < 0.05.

## Results

### Hyperphagic Behaviors and General Changes Induced by Chronic Timed Sleep Restriction

**FIGURE 1:**
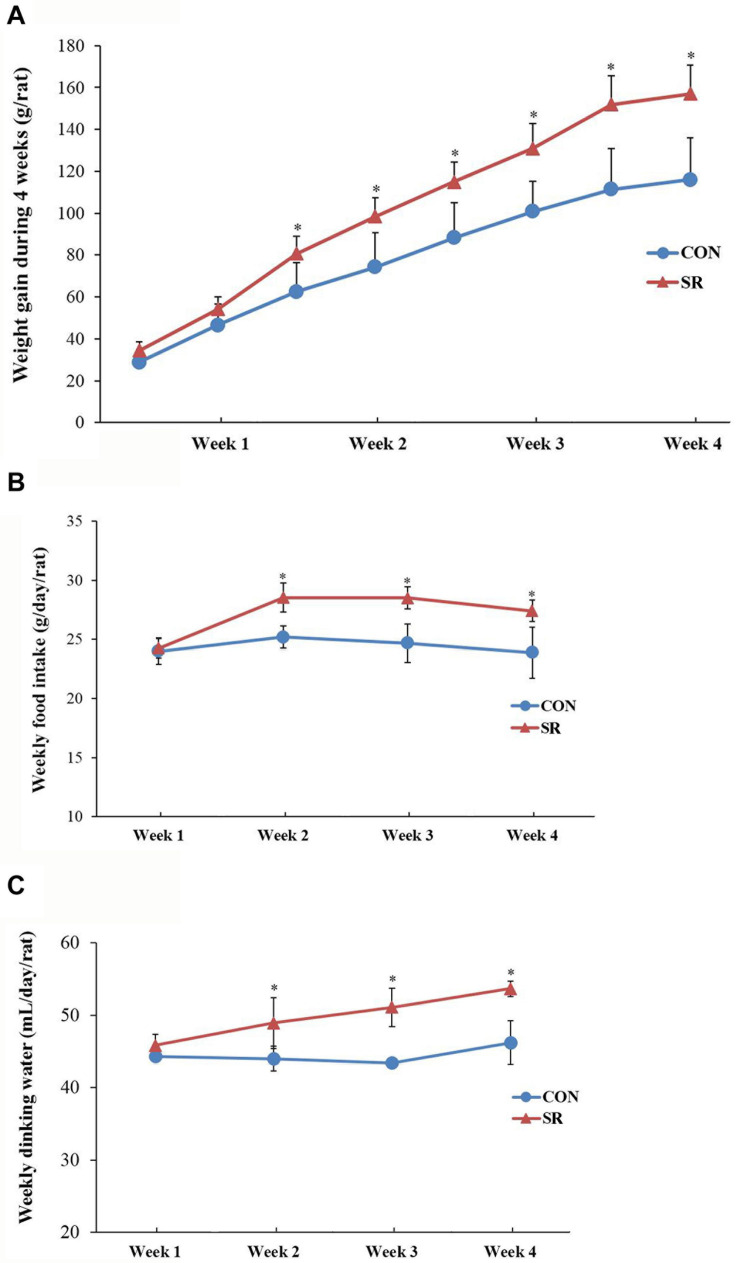
Sleep-restricted rats displayed increased weight gain and food intake. **(A)** Differences in body weight gain between the two groups after sleep restriction for 4 weeks. **(B)** Differences in food intake between the two groups after sleep restriction for 4 weeks. **(C)** Differences in drinking water intake between the two groups after sleep restriction for 4 weeks. Data were expressed as the mean ± SD. Repeated-measures analysis of variance (ANOVA) was used to compare food intake and weight gain. Significant differences were defined as *p < 0.05; *n* = 7 rats/group vs. control group. SR, sleep restriction group; CON, control group.

There were significant differences between the weight gain of rats in the control and sleep restriction groups from the second week following initiation of sleep restriction. Rats in the sleep-restricted group gained significantly more weight (*p* < 0.05, Figure 1A). The results also showed that daily ingestion of food and drinking water increased significantly in the sleep-restricted group from the second week following the initiation of sleep restriction (Figure 1B). This increased ingestion was observed throughout the remaining course of sleep restriction (*p* < 0.05, Figure 1C).

### Sleep Restriction Compromised Leptin Receptor Signaling via JAK2/STAT3 in the Hypothalamus

**FIGURE 2:**
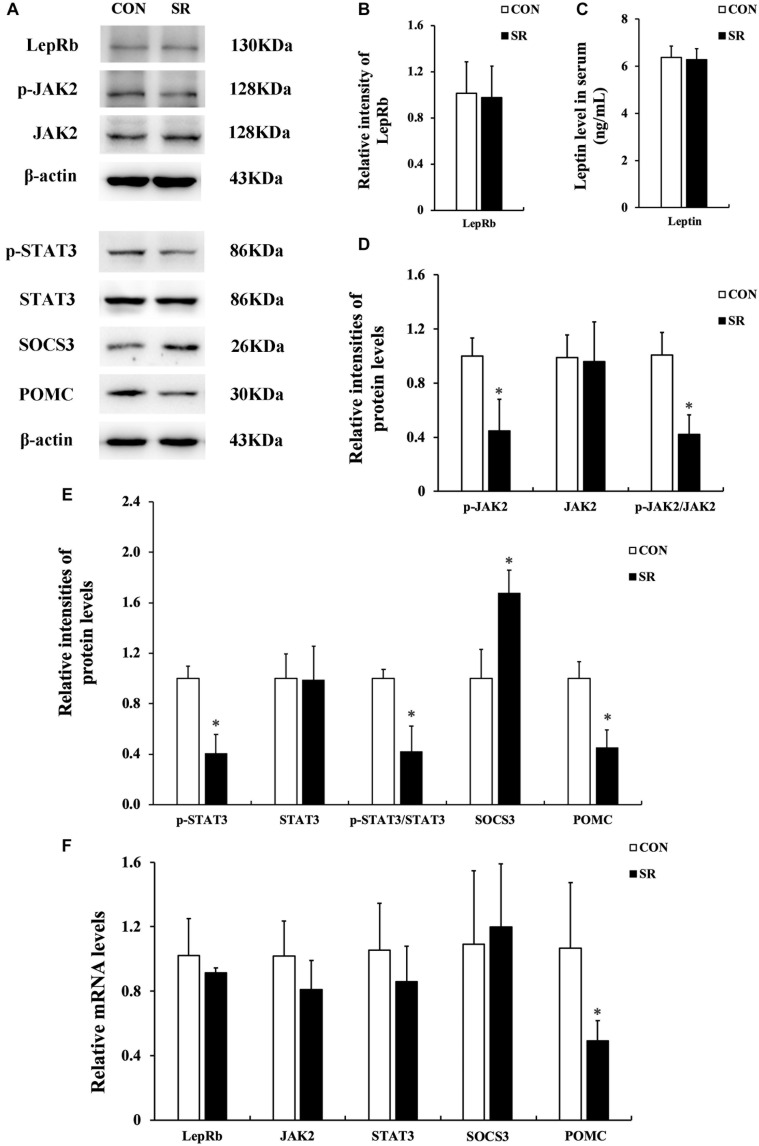
Sleep restriction compromised leptin receptor signaling via JAK2/STAT3 in the hypothalamus. **(A)** Western blot analysis. Images were the representative results of five independent experiments for each group. **(B)** Densitometric analysis of Western blots for LepRb. **(C)** Leptin levels in the serum. **(D)** Densitometric analysis of Western blots for p-JAK2, JAK2, and the p-JAK2/JAK2 ratio. **(E)** Densitometric analysis of Western blots for p-STAT3, STAT3, the p-STAT3/STAT3 ratio, SOCS3, and POMC. The relative intensity in arbitrary units compared to β-actin and presented as fold change vs. control sample. **(F)** Quantitation of LepRb, JAK2, STAT3, SOCS3, and POMC mRNA by real-time RT-PCR. Gene expression was normalized to GAPDH and presented as a fold change vs. the control. Data were expressed as the mean ± SD of six experiments and analyzed by independent-sample *t*-test. Significant difference was defined as *p < 0.05 vs. control group. SR, sleep restriction group; CON, control group.

Representative protein blots are shown in Figure 2A. We observed no difference in leptin levels in serum between the control and sleep restriction groups. Moreover, the LepRb protein levels remained unaltered in the hypothalamus during sleep restriction (Figures 2B,C). However, sleep restriction significantly reduced the protein levels of p-JAK2, p-STAT3, and POMC. This was also accompanied by reduced p-JAK2/JAK2 and p-STAT3/STAT3 ratios (*p* < 0.05, Figures 2D,E). In addition, the SOCS3 protein expression in the sleep restriction group increased significantly compared with the control group (*p* < 0.05, Figure 2E). Only POMC mRNA levels in the sleep restriction group were significantly decreased in comparison with the control group (*p* < 0.05, Figure 2F). There were no significant changes observed for any of the other genes.

### Sleep Restriction Attenuated IRS/PI3K/AKT Signaling Pathways

**FIGURE 3:**
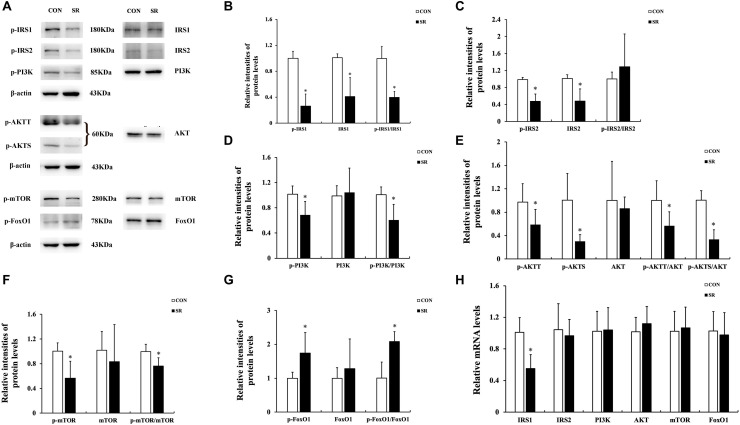
Sleep restriction attenuated IRS/PI3K/AKT signaling pathways. **(A)** Western blot analysis. Images are representative of five independent experiments for each group. **(B)** Densitometric analysis of Western blots for p-IRS1, IRS1, and the p-IRS1/IRS1 ratio. **(C)** Densitometric analysis of Western blots for p-IRS2, IRS2, and the p-IRS2/IRS2 ratio. **(D)** Densitometric analysis of Western blots for p-PI3K, PI3K, and the p-PI3K/PI3K ratio. **(E)** Densitometric analysis of Western blots for p-AKTT, p-AKTS, AKT, p-AKTT/AKT ratio, and the p-AKTS/AKT ratio. **(F)** Densitometric analysis of Western blots for p-mTOR, mTOR, and the p-mTOR/mTOR ratio. **(G)** Densitometric analysis of Western blots for p-FoxO1, FoxO1, and the p-FoxO1/FoxO1 ratio. The relative intensity in arbitrary units compared to β-actin and presented as fold change vs. control sample. **(H)** Quantitation of IRS1, IRS2, PI3K, AKT, mTOR, and FoxO1 mRNA by real-time RT-PCR. The gene expression was normalized to GAPDH and presented as fold change vs. the control. Data were expressed as the mean ± SD of six experiments and analyzed by independent-sample *t*-test. Significant differences were defined as *p < 0.05 vs. control group. SR, sleep restriction group; CON, control group.

Representative protein blots are shown in Figure 3A. The p-IRS1 and IRS1 protein expression of the hypothalamus decreased significantly in the sleep restriction group compared with the control group. This was accompanied by a reduced p-IRS1/IRS1 ratio (*p* < 0.05, Figure 3B). The p-IRS2 and IRS2 protein levels were also significantly decreased by sleep restriction (*p* < 0.05, Figure 3C). However, the p-IRS2/IRS2 ratio was unaltered in comparison with the control group. When compared to the control group, the p-PI3K, p-AKTT, p-AKTS, and p-mTOR protein levels in the hypothalamus were attenuated significantly in the sleep restriction group. This was accompanied by reduced p-PI3K/PI3K, p-AKTT/AKT, p-AKTS/AKT, and p-mTOR/mTOR ratios (*p* < 0.05, Figures 3D–F). The p-FoxO1 protein expression was increased significantly during sleep restriction compared to the control group. This was accompanied by an elevated p-FoxO1/FoxO1 ratio (*p* < 0.05, Figure 3G). Only the IRS1 mRNA level in the hypothalamus of sleep-restricted rats was decreased significantly in comparison with the control group (*p* < 0.05, Figure 3H). Other genes remained unaltered.

### Sleep Restriction Induced Depletion of Circadian Clock Gene Expression

**FIGURE 4:**
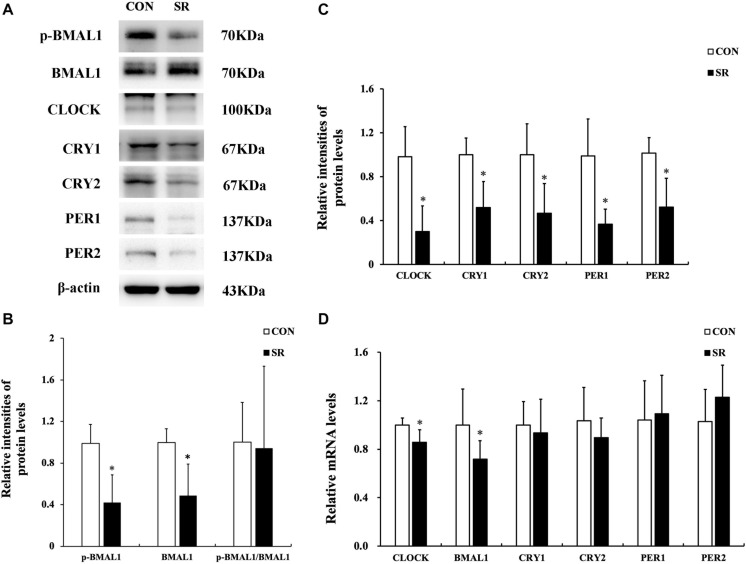
Sleep restriction induced depletion of circadian clock expression. **(A)** Western blot analysis. Images are representative of five independent experiments for each group. **(B)** Densitometric analysis of Western blots for p-BMAL1, BMAL1, and the p-BMAL1/BMAL1 ratio. **(C)** Densitometric analysis of Western blots for CLOCK, CRY1, CRY2, PER1, and PER2. The relative intensity in arbitrary units compared to β-actin and presented as a fold change vs. control sample. **(D)** Quantitation of CLOCK, BMAL1, CRY1, CRY2, PER1, and PER2 mRNA by real-time RT-PCR. The gene expression was normalized to GAPDH and presented as a fold change vs. the control. Data were expressed as the mean ± SD of six experiments and analyzed by independent-samples *t*-test. Significant differences were defined as *p < 0.05 vs. control group. SR, sleep restriction group; CON, control group.

Representative protein blots of circadian clock proteins are shown in Figure 4A. We found that both p-BMAL1 and BMAL1 protein expressions were significantly decreased during sleep restriction compared to the control group (*p* < 0.05, Figure 4B). However, this was not accompanied by a reduced p-BMAL1/BMAL1 ratio. Moreover, CLOCK, CRY1, CRY2, PER1, and PER2 protein levels in the hypothalamus were significantly reduced during sleep restriction in comparison with the control group (*p* < 0.05, Figure 4C). CLOCK and BMAL1 mRNA levels in the hypothalamus of sleep-restricted rats decreased significantly in comparison with the control group (*p* < 0.05, Figure 4D).

## Discussion

The prevalence of childhood and adolescent obesity is increasing and is a severe threat to public health worldwide. Meanwhile, the sleep duration of adolescents has continued to decline over the past 20 years (Keyes et al., 2015). It has been noted that there is a positive correlation between reduced sleep duration and elevated obesity rate (Li et al., 2017; Ogilvie and Patel, 2017; Miller et al., 2018). However, the underlying mechanisms linking sleep restriction with obesity have not been elucidated. In the present study, we first demonstrated that the weight gain and daily food intake of chronically sleep-restricted rats increased during their natural sleep course.

Secondly, we found that JAK2/STAT3/POMC signaling downstream of the leptin receptor in the hypothalamus was compromised after chronic sleep restriction, although the leptin levels in the serum and LepRb protein expression remained unaltered. The brain, especially the hypothalamus, is the master regulator of adiposity signals, maintaining energy homeostasis and body weight through the control of feeding behavior and energy expenditure. The endocrine feedback loop regulated by leptin, which is regarded as an appetite suppressant, involves leptin-producing white adipose tissue, circulating leptin, and leptin responsive in the hypothalamus, particularly the ARC (Cohen, 2006; Hopkins and Blundell, 2016; Pandit et al., 2017). Investigations performed in children and adolescents have shown that insufficient sleep duration can affect the leptin levels in the serum and affect appetite regulation, leading to weight gain, but there remain no consistent conclusions (Felsõ et al., 2017). This suggested that changes to circulating leptin levels may not be a crucial contributor to appetite regulation following chronic sleep insufficiency. Nevertheless, the response of LepRb-mediated signaling in response to serum leptin in the hypothalamus ARC might be a key influence on body weight. In the current study, dysfunction of LepRb-mediated signaling pathways in the hypothalamus was partially characterized by the compromised phosphorylation of JAK2 and STAT3, which reduced the expression of POMC protein and mRNA. Moreover, SOCS3, a leptin target gene that triggers a negative feedback loop following over-excitation of LepRb-mediated pathways, inhibits JAK2 kinase activity by directly binding to JAK2 (Pal and Sahu, 2003; Rodrigues et al., 2011; Pedroso et al., 2019). Our results showed that SOCS3 protein levels were upregulated after chronic sleep restriction, which has also previously been described by others (Hakim et al., 2015). Additionally, a previous study showed that cerebroventricular leptin infusion could increase the expression of p-STAT3 in hypothalamic regions including ARC, VMN, DMN, lateral hypothalamus (LH), and ventral premammillary nucleus (PMv), as assessed by immunocytochemistry (Sahu et al., 2013). One limitation of our study is that we used the whole hypothalamus to test the leptin responses. It will be interesting in the future to use immunohistochemistry to determine the exact hypothalamic region and cell types responsible for the leptin responses reported here.

IRS/PI3K signal pathway expression was found in the hypothalamus. IRS proteins can be recruited by JAK2, which then activate the PI3K signaling pathway (Niswender et al., 2001; Jiang et al., 2008). Previous studies have demonstrated that depletion of IRS2 and inhibition of PI3K in the hypothalamus promoted hyperphagia and obesity (Lin et al., 2004). The results drawn from the current study also showed decreased phosphorylation of IRS1, PI3K, and AKT. Furthermore, chronic sleep restriction downregulated the IRS1 expression at the transcriptional level. However, the decrease in p-IRS2 might be due to the degradation of pan-IRS2. The mTOR pathway performs a variety of physiological functions, especially serving as a cellular fuel sensor. Leptin also activates mTOR in the hypothalamus through the activation of the PI3K/AKT signaling pathway. Furthermore, POMC is one of the target genes of mTOR in the CNS (Cota et al., 2006). A recent study found that sleep deprivation could induce oxidative stress mediated by the AKT/mTOR signaling pathway in the liver of rats (Li et al., 2020). Our study is the first to highlight mTOR signaling dysfunction in the hypothalamus of chronically sleep-restricted rats. FoxO1, known as a ubiquitous transcription factor for gluconeogenesis, is an important downstream target of the PI3K/AKT pathway. FoxO1 translocates from the cytoplasm to the nucleus after phosphorylation and antagonizes the transcriptional activity of STAT3, resulting in inhibition of POMC expression and thereby increasing food intake (Kitamura et al., 2006; Sadagurski et al., 2012). In the present study, we observed the elevated phosphorylation of FoxO1 in the hypothalamus of chronically sleep-restricted rats. To conclude, we reported the reduced expression of the IRS/PI3K/AKT/mTOR and FoxO1 signaling pathways in the hypothalamus of chronically sleep-restricted rats for the first time.

Studies in humans have demonstrated that acute sleep deprivation for a continuous 24 h period can alter the epigenetic and transcriptional profile of core circadian clock genes within key metabolic tissues (Cedernaes et al., 2015). Our results showed that the protein levels of core circadian clock genes were downregulated in the hypothalamus of sleep-restricted rats, but only the mRNA levels of BMAL1 and CLOCK were decreased. The transcription factors BMAL1 and CLOCK usually exist as heterodimers and induce the expression of the repressors CRY1, CRY2, PER1, and PER2 via E-box sequences in their promoters. CRYs and PERs translocate into the nucleus and suppress CLOCK:BMAL1-mediated transcription after translation, thereby forming a negative feedback loop (Vogel et al., 2015; Bravo Santos et al., 2016; Rácz et al., 2018). Therefore, the reduction of BMAL1 and CLOCK mRNA levels observed in our study could be the initial contributors to depletion of circadian clock expression in the hypothalamus. This was perhaps directly or indirectly caused by chronic timed sleep restriction. The circadian clocks in the SCN of the hypothalamus intensify the responses of LepRb to serum leptin in the ARC to maintain the balance of energy metabolism. Chronic circadian rhythm dysfunctions can lead to desensitization of LepRb to circulating leptin, thereby causing leptin resistance (Kettner et al., 2015). In accordance with the above, the current study revealed that depletion of circadian clock expression might occur in parallel with the compromise of LepRb-mediated signaling pathways, but the interactions between these two phenomena could not be confirmed and require further exploration. Another limitation of the current study was that electroencephalograph (EEG) and electromyography (EMG) recording was not applied to monitor the sleep course, so that the amount of sleep that the rats actually had could not be accessed. We plan to use EEG/EMG recording in the future study to accurately access sleep structure.

In summary, our present study might unveil a novel insight for the underlying mechanisms involved in appetite regulation in the hypothalamus following chronic timed sleep restriction in adolescent to young adult rats. We demonstrated that both LepRb-mediated JAK2/STAT3 and IRS/PI3K/AKT signaling pathways were compromised after chronic timed sleep restriction for 4 weeks and occurred in parallel with depletion of circadian clock gene expression.

## Data Availability Statement

The raw data supporting the conclusions of this article will be made available by the authors, without undue reservation, to any qualified researcher.

## Ethics Statement

The animal study was reviewed and approved by the Animal Use and Care Committee of China Medical University, abiding by National Institutes of Health Guide for Care and Use of Laboratory Animals.

## Author Contributions

QS, LJ, and DeW designed the research. QS, YL, and WW participated in the data collection. QS and LJ wrote the manuscript. QS, DeW, and RL performed the data analysis. All the authors read and approved the final manuscript.

## Conflict of Interest

The authors declare that the research was conducted in the absence of any commercial or financial relationships that could be construed as a potential conflict of interest.
